# Walk the plank! Using mobile electroencephalography to investigate emotional lateralization of immersive fear in virtual reality

**DOI:** 10.1098/rsos.221239

**Published:** 2023-05-31

**Authors:** Yasmin El Basbasse, Julian Packheiser, Jutta Peterburs, Christopher Maymon, Onur Güntürkün, Gina Grimshaw, Sebastian Ocklenburg

**Affiliations:** ^1^ Department of Biopsychology, Faculty of Psychology, Institute of Cognitive Neuroscience, Ruhr-University Bochum, Universitätsstrasse 150, 44780 Bochum, Germany; ^2^ Netherlands Institute for Neuroscience, Social Brain Lab, 1105 BA Amsterdam, The Netherlands; ^3^ Institute for Systems Medicine & Department of Human Medicine, MSH Medical School Hamburg, Victoria University of Wellington, Wellington 6140, New Zealand; ^4^ School of Psychology, Victoria University of Wellington, Wellington 6140, New Zealand; ^5^ Research Center One Health Ruhr, Research Alliance Ruhr, Ruhr University Bochum, Bochum, Germany; ^6^ Department of Psychology, MSH Medical School Hamburg, Am Kaiserkai 1, 20457 Hamburg, Germany; ^7^ Institute for Cognitive and Affective Neuroscience, Medical School Hamburg, Am Kaiserkai 1, 20457 Hamburg, Germany

**Keywords:** mobile electroencephalography, emotional lateralization, virtual reality, emotion induction, ecological validity, international affective picture system

## Abstract

Most studies on emotion processing induce emotions through images or films. However, this method lacks ecological validity, limiting generalization to real-life emotion processing. More realistic paradigms using virtual reality (VR) may be better suited to investigate authentic emotional states and their neuronal correlates. This pre-registered study examines the neuronal underpinnings of naturalistic fear, measured using mobile electroencephalography (EEG). Seventy-five healthy participants walked across a virtual plank which extended from the side of a skyscraper—either 80 storeys up (the negative condition) or at street level (the neutral condition). Subjective ratings showed that the negative condition induced feelings of fear. Following the VR experience, participants passively viewed negative and neutral images from the international affective picture system (IAPS) outside of VR. We compared frontal alpha asymmetry between the plank and IAPS task and across valence of the conditions. Asymmetry indices in the plank task revealed greater right-hemispheric lateralization during the negative VR condition, relative to the neutral VR condition and to IAPS viewing. Within the IAPS task, no significant asymmetries were detected. In summary, our findings indicate that immersive technologies such as VR can advance emotion research by providing more ecologically valid ways to induce emotion.

## 1. Introduction

Since early hints from lesion research [1–3], many studies have supported the notion that emotion processing in the brain is lateralized [4]. Despite the consensus that emotions are processed asymmetrically in the brain, an exhaustive theory explaining the underlying mechanisms is yet to be found. Rather, there are several theories each holding strong empirical support for different aspects of emotional experience but also showing considerable inconsistencies. For example, the two most-established theories about emotion lateralization are the right hemisphere and the valence hypothesis. The former postulates that the right hemisphere is responsible for emotional experiences irrespective of valence [5–10], whereas the latter differentiates between the valence of the emotion, with positive emotions being processed in the left hemisphere and negative emotions being processed in the right hemisphere [11–15]. To this day, studies gather evidence for both theories, even though the core predictions are not complementary [16].

One reason for these mixed findings could be the lack of ecological validity in the operationalization of emotions. In laboratory settings, the most popular tools to elicit emotions experimentally are images (e.g. international affective picture system (IAPS) [17]) or emotional films. Adding moving pictures should increase immersion and saliency of the stimuli, thereby affecting emotions more strongly than static images [18]. In general, both images and films were shown to elicit behavioural and physiological emotional reactions (images [19,20]; films [21,22]). However, their repetitive presentation in block designs conflicts with an ecologically valid understanding in which emotions are characterized by their spontaneous and phasic nature. Moreover, the mere perception of emotional stimulus material does not reflect the multi-faceted nature of emotional experience. In real life, perceiving emotional content quickly translates into and prepares the body for action like the fight-or-flight response [23]. These crucial components of emotion are neglected by studies focusing solely on emotion perception. Although a recent study on naturalistic disgust induction confirmed such notions, the presentation of pictures was still highly effective in inducing disgust, highlighting that pictures are still a valid option to induce emotions [24]. Since a more naturalistic setting was nonetheless more effective in this study, aiming for high ecological validity to study emotional processing is likely to be advantageous.

One potential method to increase ecological validity is the use of virtual reality (VR). VR is already widely used for treatment in clinical populations, for example, for exposure therapy in fear of heights [25], social phobia [26,27] or eating disorders [28]. In healthy participants, VR is often used for educational purposes [29–31] or to investigate spatial navigation [32–34]. One of the major advantages of VR is the possibility to simulate real-life situations realistically while minimizing external influences. Thereby, VR bridges experimental control and ecological validity. Because the environment responds to participants' movements, VR offers a higher degree of immersion into the experimental paradigm compared with two-dimensional content, making it effective in eliciting ‘authentic’ emotions [35–37]. Fear induction, particularly, can benefit from more realistic methods like VR. Although fear has been successfully elicited in prior experiments, as evident in self-report and bodily reactions like elevated heart rate or skin conductance [19,20], the selection of suitable (but also ethical) stimulus material or situations can be challenging. For example, Barke *et al.* [38] found that some IAPS images selected by experts to evoke fear (especially images depicting human threat) actually provoked anger in the participants. Moreover, fear evoked by pictures does not have the intensity of genuine threat. By using interactive simulations resembling fearful situations in real life, VR could offer a more valid approach to experimental fear induction. This in turn could further widen the scope of emotion research from mere perception to perception-to-action. It needs to be noted, however, that use of VR does not necessarily lead to stronger emotion induction compared with mere visual presentation on a screen. For example, van der Wal *et al*. [39] compared the effectiveness in fear induction using two-dimensional video clips and did not find any differences between these two presentation methods. Thus, the effectiveness of VR in contrast with a simple picture or video clip should be assessed carefully prior to study onset, especially given the higher economic cost and potential increased discomfort [40] associated with VR use.

In order to investigate the neuronal underpinnings of emotional experience, VR can be combined with mobile electroencephalography (EEG) systems [41,42]. These systems possess acceleration sensors specifically designed to take movement into account which can then be integrated in data analysis. Thereby, a major drawback of stationary EEG studies for emotion research can be addressed. In a conventional EEG system, the electrodes are connected to the amplifier via cables, so that it is usually necessary that participants lay their head on a chin rest to avoid movements as much as possible. This, however, severely limits the spectrum of paradigms suitable for EEG experiments [43] particularly affecting emotion research. Natural spontaneous reactions such as frowning, smiling, wincing or body movements which are indicative of experiencing real emotions become disadvantageous for later data analysis and are sought to be suppressed. By contrast, the largely wireless signal transmission of mobile EEG systems allows for free movement without notable interference with the neuronal signal [44–46], meaning that researchers can take advantage of both the heightened perceptual experience in VR and the freedom of movement it affords.

In the present pre-registered study, we aim to investigate the neuronal correlates of experimentally induced fear in two paradigms with varying levels of ecological validity. The first task comprises a naturalistic VR setting with high ecological validity. Here, participants balance either on a virtual plank on a tall skyscraper (negative condition) to induce fear of heights, or on the ground (neutral condition). The second task comprises a typical fear induction paradigm with low ecological validity during which the participants are presented with fearful or neutral IAPS images. As both major theories in emotional lateralization (right hemisphere and valence hypothesis) predict a stronger right-hemispheric activation in situations of negative valence, we predict stronger rightward activation in both tasks. Since stronger experiences of emotions are furthermore associated with higher levels of asymmetry for negative emotions [47], the more realistic fear evoked by the VR task could also be reflected in a more lateralized rightward activation pattern compared with the non-realistic IAPS task.

Based on previous research on emotion processing, hemispheric asymmetries are quantified using alpha power. Alpha power can be derived from the EEG recording and is inversely correlated with cognitive activity. Higher alpha power on a given electrode site indicates relatively less activity in that region [48]. Since emotional content requires attentional and cognitive resources, alpha power is expected to decrease as a function of emotional intensity. While previous research has also suggested that asymmetries in other frequency bands such as beta and gamma power are involved in the processing of emotions [49,50], we chose to focus on alpha power as the experimental findings on alpha power, especially on frontal electrodes, have been most consistent in the literature of emotional lateralization. To deduce hemispheric asymmetries, alpha power from homologous regions in both hemispheres can be compared using the asymmetry index, particularly for frontal electrode pairs F3/F4 [51,52] and F7/F8 [44]. Complementing the neurophysiological measures, fear ratings are acquired during the VR task.

We predict that alpha at F3/F4 and F7/F8 will show greater relative left-sided power in the negative VR condition compared with the neutral VR condition. This would reflect right-hemispheric processing of negative emotion as predicted both by the right hemisphere and valence hypotheses. Similarly, greater left than right alpha power at the electrode pairs F3/F4 and F7/F8 is predicted for the negative condition of the IAPS task compared with the neutral condition. It will be explored whether alpha asymmetries in the VR task are more pronounced overall than in the IAPS task as a response to the more realistic fear exposure. If alpha asymmetry supports fear experience, we further predict that the self-reported levels of fear should positively correlate with higher left-sided alpha power indicative of stronger right-hemispheric activation. Finally, we predict that alpha power asymmetries should show higher correlations during the neutral conditions of both tasks due to similar (non)-emotional processing, relative to the negative conditions of both tasks, given the expected difference in negative emotions due to differences in ecological validity. All hypotheses and analyses were pre-registered at Open Science Framework (https://osf.io/uv26f).

## 2. Material and methods

### 2.1. Participants

We aimed to collect data from 80 participants prior to data analysis. A power analysis of a within-participant repeated-measures ANOVA with three measurements and four groups revealed that in this sample size, it is possible to detect small-to-medium sized effects (Cohen's *f* = 0.16) at 80% power. Ninety-three potential participants filled out an online survey to take part in the experiment. Exclusion criteria were age (below 18 and above 35 years), diagnosis of mental/neurodevelopmental disorders or neurological diseases (e.g. migraine, epilepsy) and extreme fear of heights. One person did not fit the age criterion and thus could not participate, and one person was excluded *a posteriori* due to being older than 35 years. There were four cases in which mental disorders led to an exclusion from the study, namely one person whose score on the Beck Depression Inventory [53] was above the cut-off value (51; a value greater than or equal to 30 indicates severe depression) and three persons reporting bipolar disorder, panic disorder, and depression with addiction. These measures were taken due to empirical evidence revealing altered hemispheric asymmetries in clinical samples compared with non-clinical samples [54,55]. For the same reason, high values in the Acrophobia Questionnaire (ACRO/AVOI [56]; German version [57]) were considered an exclusion criterion which affected one potential participant. After applying the exclusion criteria, the sample comprised 87 healthy participants (54 women, 33 men) with normal or corrected-to-normal vision. In a second step, participants were excluded due to issues during data acquisition or data analysis. In one case, the experiment had to be cancelled during the VR condition, because the participant reported sudden anxiety symptoms and could not complete the task. In two cases, technical difficulties with the IAPS paradigm occurred leading to exclusion from data analysis. EEG pre-processing revealed missing triggers in four and incorrect markers in two EEG recordings, which also led to the exclusion of these files from further analysis. These technical issues were probably caused by signal loss in the connection of the mobile EEG system to the recording laptop. One person was excluded from analysis because the screening questionnaire was not filled out. Thus, the final sample used for statistical analysis comprised 75 participants (45 women, 30 men) with a mean age of 24 (s.d. = 3.61). A sensitivity analysis showed that this sample retained the ability to detect small to medium effects (Cohen's *d* = 0.34) at 80% power.

The study was accepted by the ethics committee of the Faculty of Psychology at Ruhr-University Bochum. All participants gave their written informed consent at the beginning of the experiment. The experiment was conducted in accordance with the Declaration of Helsinki.

### 2.2. Stimulus material

#### 2.2.1. International affective picture system paradigm

Two sets of stimuli were chosen from the IAPS database (https://csea.phhp.ufl.edu/index.html), one consisting of 50 neutral images and one with 50 negative images. Negative images were picked according to their arousal and valence ratings determined by previous validation studies [17,58]. In the negative sample, only images with an arousal rating over 4.3 (*M* = 6.33; s.d. = 0.67) and a valence rating under 2.54 (*M* = 2; s.d. = 0.41) were included to ensure that the pictures reliably induce negative affect. Negative stimuli mostly contained pictures displaying mutilation, injuries and violent scenes. The neutral stimulus set was composed of pictures showing mainly household objects, nature and different patterns. The IAPS conditions were programmed using the software Presentation.

#### 2.2.2. Virtual reality paradigm

The task with high ecological validity for fear induction was presented using VR. The VR simulation was presented using an HTC Vive head-mounted display (HMD) with corresponding hand controllers (https://www.vive.com/us/). The HTC Vive has an inbuilt display with a 2160 × 1200 pixel resolution, a 110° field of view and a refresh rate of 90 Hz. It furthermore features accelerometers, a gyroscope and adjustable lenses. The VR environment was created in the laboratory using the Unity game engine (https://unity.com/). The simulation was projected to the HMD using the SteamVR [59] runtime application.

**Figure 1.**
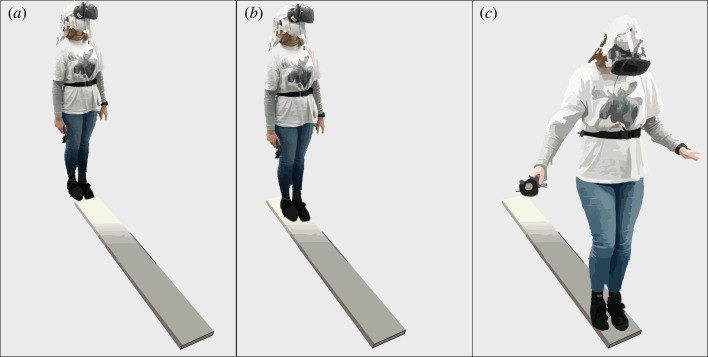
Recording segments during the plank task. (*a*) The first recording started when the participants had positioned themselves in front of the plank (elevator). In the VR environment, the view of the participant would have been from within the elevator either on the street level (neutral condition) or at a high elevation (negative condition). (*b*) The second recording segment took place after the participants took a step onto the plank (start of plank). (*c*) The final recording segment was taken at the end of the plank (end of plank).

**Figure 2.**
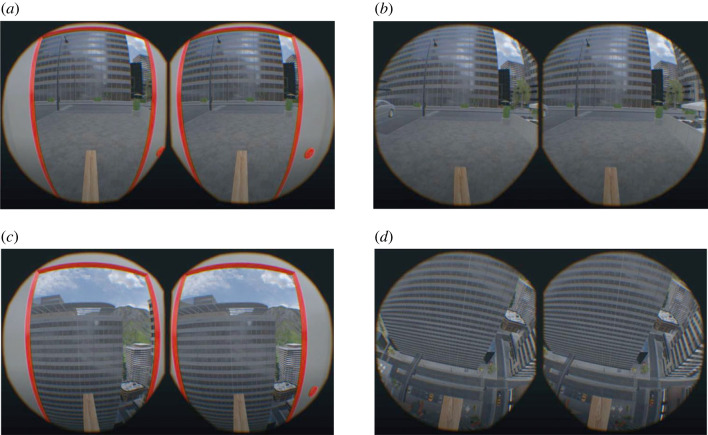
Example views from the participants' perspective. (*a*) Street view from inside the elevator in the neutral condition. (*b*) Street view in the start of plank recording segment in the neutral condition. (*c*) View from the building top from inside the elevator in the negative condition. (*d*) Downwards view from the end of plank recording segment in the negative condition.

The physical measurements of the virtual room were approximately 4 × 2.5 m. The VR set-up included two base stations (height: *ca* 2.4 m, distance between the base stations: *ca* 3 m) framing the virtual room, the HMD, two controllers and one laptop with an external number pad to choose VR conditions. The controllers transformed into the participants' hands while in the virtual environment. The laptop showing the VR paradigm was a Dell laptop (Intel(R) Core(TM) i7-9850H CPU @ 2.60 GHz) running Windows 10 with an NVIDIA Quadro T2000 graphics card. Apart from that, there was one laptop to display the IAPS task. One additional laptop was needed for EEG signal recording. The VR paradigm looked as follows:

Upon starting the VR set-up, participants were projected into the virtual room represented by a city environment. The participants started at ground level afoot of a tall skyscraper inside its elevator. The door of the elevator was open to provide the participants with an unobstructed street view. Participants were asked to step outside the elevator slowly moving towards the street. The participants then turned around and re-entered the elevator. Participants then used their right hand controller to press a red button on the right side of the elevator door, causing the elevator doors to close and the elevator to move upwards. In the neutral control condition, the elevator moved upwards only for a short period before returning to the street level again. In the negative experimental condition, the elevator continued to the top of the building. Opening of the elevator doors marked the beginning of a 1 min segment in which the participant was told to look around without walking or talking (figure 1*a* depicts the experimenter perspective, figure 2*a* depicts the participant perspective for the neutral condition and figure 2*c* depicts the participant perspective for the negative condition). The manual EEG trigger was pressed to set accurate time points for subsequent analyses. After 1 min, the experimenter asked for the first rating (‘elevator’ rating). Next, the participants took one step onto the plank and stopped there, initiating the next 1 min interval of looking around (figure 1*b* depicts the experimenter perspective, and figure 2*b* depicts the participant perspective for the neutral condition). Having completed 1 min, participants were asked to give their second rating (‘start of plank’ rating). Afterwards, participants were instructed to walk further along the plank until the end of the plank was reached (figure 1*c* depicts the experimenter perspective, and figure 2*d* depicts the participant perspective for the negative condition). Stopping there marked the beginning of the third 1 min long recording interval of the EEG. After 1 min, participants gave their rating again (‘end of plank’ rating). Participants could then walk back on the plank and re-enter the elevator thereby finishing the respective condition. Apart from the virtual environment, the experimental condition and control condition followed an identical protocol.

It needs to be noted that the experimental paradigm employed in the study deviated from the outlined design in the pre-registration. In the pre-registration, six time points for EEG recordings were planned (at street level, bottom of elevator, top of elevator, on plank, end of plank, turning back). These were reduced to three, as two of these conditions would have had complete overlap between the experimental and control condition (street level and bottom of elevator). The turning back condition was removed to avoid a condition with considerable movement artefacts as participants would have manoeuvred on the plank.

### 2.3. Behavioural measures

#### 2.3.1. Questionnaires

Potential participants were sent a link to an online screening on the platform Qualtrics (https://www.qualtrics.com/) for relevant exclusion criteria. The screening contained demographic information, exclusion criteria and a COVID-19 risk and symptom screening. In terms of demographic information, age, gender, vision, spoken languages, educational achievement and mental and physical health were enquired. Furthermore, various questionnaires relevant for later analyses were included. The State-Trait Anxiety Inventory-Trait (STAI-T [60]) measures self-reported trait anxiety with 20 items. On a 4-point Likert scale (from ‘not at all’ to ‘very much so’), participants determine to what extent they generally agree with different anxiety-related statements (e.g. ‘I feel rested.’) resulting in a trait anxiety score from 20 to 80. The Acrophobia Questionnaire (ACRO/AVOI [57]) assesses the self-reported extent of fear (ACRO) and avoidance (AVOI) of 20 specifically height-related situations (e.g. standing on the edge of a railway track, riding a Ferris wheel or standing on a balcony on the 10th floor). For each situation, participants report how anxious they would feel given that situation and to what extent they would avoid it on a 7-point Likert scale (from ‘not at all anxious/would not avoid doing it’ to ‘extremely anxious/would not do it under any circumstances'). Consequently, a mean score can be calculated each for fear and avoidance of heights. It is important to note that the ACRO/AVOI was not designed to differentiate between clinically relevant acrophobia and non-clinical unease of heights. After completing the screening, participants were contacted via e-mail to arrange an appointment for the experiment.

On site, participants filled out the state version of the STAI (STAI-S [60]). The STAI-S resembles the trait version (STAI-T) regarding the items but assesses state anxiety in that given moment. This way, baseline anxiety before experimental fear induction was documented. To assess overall affective states, the Positive and Negative Affect Schedules (PANAS, German version [61]; English version [62]) was given to the participants. The PANAS comprises 20 adjectives describing different feelings and moods (e.g. strong, worried, or proud). On a 5-point Likert scale from ‘not at all’ to ‘extremely’, participants tick the box which best describes their feelings in that moment.

Finally, the Simulator Sickness Questionnaire (SSQ) [63] and the iGroup Presence Questionnaire (IPQ) [64] were filled out by the participants. These questionnaires evaluate to what extent participants felt immersed and affected by the VR simulation. The SSQ evaluates on a 4-point Likert scale (from ‘not at all’ to ‘strongly’) to what degree participants experienced 16 symptoms characteristic for simulations in VR, e.g. blurred vision, dizziness (with eyes open or closed) or general discomfort. The Sense of Presence Inventory assesses how present participants felt in the virtual environment and if a sense of ‘being there’ [64] could be induced. To do so, participants were asked, for example, how aware they were of the real world while being in the virtual world.

#### 2.3.2. Affective ratings

Two kinds of ratings were carried out addressing subjective fear and presence. Ratings were given within a range from 1 (not fearful/present) to 10 (extremely fearful/present) at three recording segments (elevator, start of plank and end of plank). Before entering the VR simulation, a test fear rating was implemented to check whether participants had understood the ratings. This test rating was not included in the statistical analysis.

### 2.4. Physiological measures

**Figure 3.**
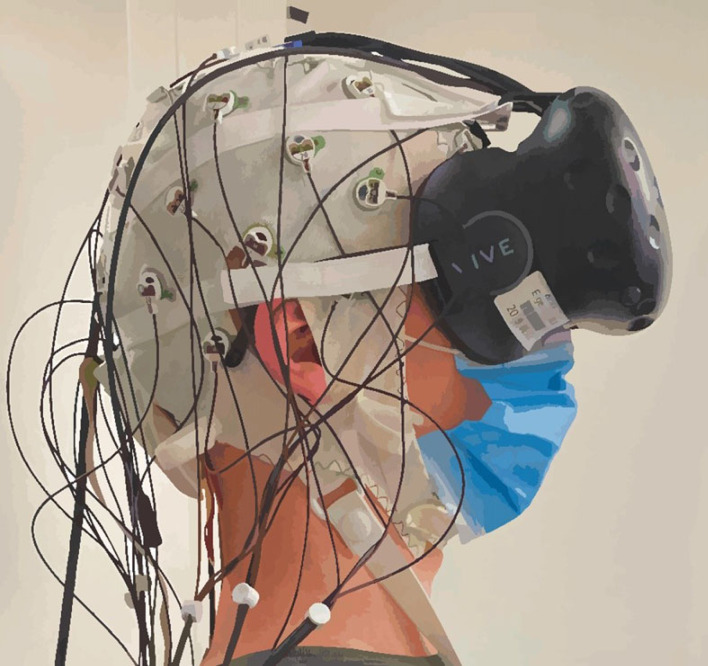
Set-up of the EEG recording and VR system on the participant. The electrode cap was fitted first before the VR system was mounted on the participant's head. The straps holding the VR system in place were carefully placed in-between the electrodes.

Brain activation was measured with a mobile EEG system (LiveAmp 32, Brain Products GmbH, Gilching, Germany). This system consists of 32 electrodes arranged in line with the international 10–20 system [65]. The position of the Fpz electrode was set as the ground electrode, and FCz served as the reference electrode. A wireless amplifier amplified the recorded activation transmitting it to the recording software (Brain Vision Recorder) on a laptop with a sampling rate of 1000 Hz. The impedance cut-off was set to less than 10 kΩ for an adequate EEG signal. Three acceleration sensors implemented in the wireless amplifier measured head and body movements along the *X*-, *Y*- and *Z*-axes to enable subsequent movement correction in the recordings. Several EEG caps in different sizes were adjusted for VR-compatible use by MES Forschungssysteme GmbH (https://mes.gmbh/). To this end, five Velcro straps were sewn onto each cap, attaching the HMD without interfering with the electrodes (figure 3).

In the pre-registration, we also noted that we wanted to measure heart rates in the participants as a further outcome variable. To this end, we used wrist watches (Polar heart rate monitor watches, https://www.polar.com/) that were worn by the participants during the entire experimental protocol. Unfortunately, heart rate data were not consistently transmitted by the watches, resulting in a large number of missing values. Furthermore, *post hoc* inspection of the data did not reveal any fluctuations in heart rate across the individual experimental periods, which calls their validity into question. We, therefore, deviated from the pre-registration and did not analyse heart rate data.

### 2.5. Procedure

Participants were tested in a designated test room in the Biopsychology department at Ruhr**-**University Bochum. The duration of the experiment was approximately 2 h. Due to the ongoing COVID-19 pandemic at the time of the inquiry, participants were obliged to undergo a COVID-19 self-test and to wear a mask ensuring minimal infection risk for both participant and experimenter. The participants were then asked to fill out the written informed consent, a COVID-19 symptom questionnaire and an attendance list to track contacts in the event of a positive COVID-19 case. The LiveAmplifier was placed in a small pocket at the back of the EEG cap. After a secure wireless connection between LiveAmplifier and recording laptop was established, the EEG cap was prepared with electrode gel to allow signal detection and transmission. To connect the LiveAmplifier with the Sensor and Trigger Extension box, participants wore a belt bag containing the latter and a power bank to guarantee power supply of the equipment. The first EEG recording captured resting state EEG while sitting. Participants were asked to close their eyes for 5 min and relax without concentrating on anything particular. During this time, the experimenter left the room and turned off the lights to avoid distraction. After 5 min elapsed, participants filled out the first PANAS and the STAI-S questionnaire.

To prepare for the VR task, a wooden plank (width: 19 cm; depth: 4 cm; length: 151 cm) was placed in the centre of the room. The plank was manipulated so that it was slightly unstable, wobbling to the left and right at each step. Before entering VR, participants had the chance to walk on the plank once to eliminate novelty effects. Thereafter, participants were introduced to the two affective ratings (presence in VR and subjective fear) enquired during the VR task. The experimenter asked for the first rating to check if the ratings were understood correctly. Subsequently, the HMD was fitted to the EEG cap and the participant was guided to the starting point of the VR task. The order of the negative and neutral condition was counterbalanced. After ensuring that the simulation started properly, the participants were handed the two controllers. To enhance presence in the VR, they were told to make themselves familiar with them by operating their virtual hands and fingers. The experiment then followed the procedure outlined in 2.2.2.

**Figure 4.**
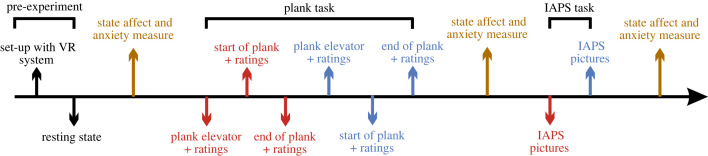
Timeline of the experimental procedure. Before the experiment, participants first went through a resting state before providing information about state affect and anxiety. After being set up with the VR equipment, the plank task was conducted. Note that the red and blue colours illustrate the negative and neutral conditions which were counterbalanced across participants. Ratings include the presence as well as subjective fear rating. After completing the plank task, another state affect and anxiety measure were taken. The following IAPS task also comprised a negative and neutral condition (counterbalanced across participants). Finally, state affect and anxiety were measured before the end of the experiment.

Having completed the VR task, the HMD was loosened from the EEG cap. Participants could take a seat to fill out the second PANAS questionnaire and to do the second part of the experiment. The manual trigger was unplugged from the trigger box and the latter was connected to the presentation laptop so that automatic triggers embedded in the IAPS presentation could be transmitted to the recording laptop. The proper IAPS condition was prepared following the counterbalanced order, starting either with neutral or with negative pictures. The participants were briefed to observe the pictures attentively and thoroughly without looking around the room. The presentation started by pressing the spacebar on the presentation laptop. Simultaneously, the experimenter ran the new EEG file. During the presentation, the experimenter left the room and turned the lights off to avoid distraction. Each picture was displayed for 5 s with an interstimulus interval of 2 s, leading to a total trial duration of 7 min for each condition. Directly following the first set of pictures, the second set was started, and the procedure described above was repeated. When the second set was finished, participants were instructed to fill out the post-experiment survey via Qualtrics, including the third PANAS [61], the SSQ [63] and the IPQ [64]. Subsequently, the EEG equipment (i.e. belt bag, EEG cap) was removed from the participants, and they were compensated either with 20€ or course credit. The timeline of the experiment is illustrated in figure 4.

### 2.6. Electroencephalography pre-processing

For data pre-processing, the program BrainVision Analyzer (Brain Products GmbH, Gilching, Germany) was used. First, the sampling rate was adjusted from 1000 to 250 Hz with a sampling interval of 4000 µs to shorten processing time. A 0.5 Hz high-pass filter (zero phase shift Butterworth filter, time constant 0.3183099) was applied to avoid interference from non-physiological high-frequency sources. To exclude noise from electronic devices in the experimental setting, a 50 Hz notch filter was used. First, electrodes that showed notably low (i.e. flat lines) or high (i.e. unusual spikes) variance were removed from the recording. Then, these channels were interpolated using the surrounding electrodes. Raw data were inspected manually, detecting and removing artefacts caused by coughing or excessive movement. An infomax independent component analysis extracted pulse and eye artefacts (i.e. blinking and horizontal eye movement). The reference electrode FCz was interpolated. The data from the plank task were then segmented according to the three markers (S1: elevator; S2: start of plank; S3: end of plank) set with the manual trigger. Segment length of the first two markers varied, since each following marker constituted the end of the prior interval (e.g. S2 marked the end of the segment starting with S1). The segment period for the third marker was 60 000 ms. Overlap of segments was not allowed. The average of all channels was then set as the new reference (Avg). A second filter was applied with a low cut-off at 1 Hz and a high cut-off at 45 Hz. Segments with a length of 1 s were created via segmentation. A fast Fourier transformation (Hanning window of 10%) was applied to extract alpha oscillations (8–12 Hz) from the signal. Lastly, the average power was calculated for each recording segment in the plank conditions. In the IAPS task, the pre-processing steps were the same as for the plank task except that there were no segments following marker positions. Segments of 1 s were created for the whole length of the paradigm regardless of stimulus markers. Consequently, only one average was calculated for the whole IAPS condition for later power analysis.

For movements, no initial infinite impulse response (IIR) filter was applied to enable analysis of the raw data. All electrodes except for the three acceleration sensors were disabled. In the plank task, data from the acceleration sensors were segmented following the same logic based on the marker positions of S1, S2 and S3 described above. In a second step, segments with a length of 1 s were defined. For each recording segment, an average was calculated. Data from each acceleration sensor (*X*-axis, *Y*-axis and *Z*-axis) were exported separately for the recording segments. The same procedure was applied to the movements in the IAPS task. There was again one average for the whole condition from which the data for the acceleration sensors were exported separately.

To investigate hemispheric asymmetries in the experiment, the asymmetry index was calculated for the electrode pairs F3/4 and F7/8. The asymmetry index (AI) is obtained by subtracting the natural logarithm of the frequency band power of a specific left-hemispheric electrode site from the natural logarithm of the frequency band power of its right-hemispheric homologue [66]. The formula is as follows:

AI=ln(power right)−ln(power left).

A positive alpha index indicates greater relative left than right activation and a negative alpha index indicates greater relative right than left activation as alpha is inversely correlated with brain activity [48].

### 2.7. Statistical analyses

#### 2.7.1. Pre-registered analyses

*Hypotheses 1 and 2*

A one-group repeated-measures design was used with the within-participant factors condition (negative, neutral), validity (plank, IAPS) and electrode pair (F3/F4, F7/F8). Asymmetry indices (AIs) served as dependent variable. Therefore, a 2 × 2 × 2 repeated-measures ANOVA with the described factors was calculated. For the plank task, a repeated-measures 2 × 2 × 3 ANOVA with the within-participant factors valence (neutral, negative), electrode (F3/4, F7/8) and recording segment (‘elevator’, ‘start of plank’, ‘end of plank’) was applied. This analysis deviated from the pre-registration since the original experimental design in the pre-registration planned for six recording segments (at street level, bottom of elevator, top of elevator, on plank, end of plank, turning back) instead of three that were employed in the final experiment (top of elevator, start of plank, end of plank). Thus, the pre-registration originally planned a 2 × 2 × 6 ANOVA. Since the experimental protocol was changed prior to data collection, the analysis design was adapted accordingly. Repeated-measures ANOVAs were Greenhouse–Geisser corrected if sphericity was violated. Although normality was often violated in our outcome variables (electronic supplementary material, table S1), we used parametric testing because repeated-measures ANOVAs, especially with a high number of data points, have been demonstrated to be robust against normality violations. Significant main and interaction effects were corrected *post hoc* using the Bonferroni method.

*Hypotheses 3 and 4*

Self-reported fear ratings were correlated using Pearson correlations with the AIs in the corresponding VR condition and recording segment. AIs of the neutral and the negative IAPS condition were correlated with the AIs of the neutral and negative plank condition.

#### 2.7.2. Exploratory analyses

*Self-report data*

For the VR task, a 2 × 3 repeated-measures ANOVA of fear ratings with the within-participant factors valence (neutral, emotional) and recording segment (‘elevator’, ‘start of plank’, ‘end of plank’) was calculated.

Fear ratings were correlated with the ACRO/AVOI scale, the trait and state version of the STAI, the SSQ and with presence ratings. Presence ratings were correlated with the IPQ from the screening survey.

A repeated-measures 3 × 2 ANOVA of the three PANAS questionnaires with the within-participant factors time (baseline, post-VR, post-IAPS) and affect (positive, negative) was conducted.

*Movement signals*

For every condition (neutral, negative) and electrode pair (F3/4, F7/8), a multiple linear regression with the AI as dependent variable and the individual acceleration sensor signals (*X*, *Y* and *Z*) as predictors was calculated to detect influences of movement on the EEG signal. In the plank task, every recording segment was checked separately. The assumption of independent errors was tested using the Durbin–Watson statistic. Analyses were checked for collinearity and normality using the variance inflation factor (VIF) and P-P-plots.

## 3. Results

### 3.1. Pre-registered analyses

#### 3.1.1. Hypotheses 1 and 2

**Figure 5.**
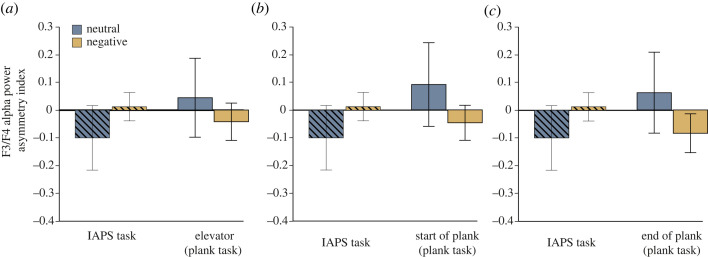
Asymmetry indices according to task for the neutral and negative conditions at the F3/F4 electrode sites. (*a*) Comparison between IAPS results (hatched) and the ‘elevator’ segment from the plank task. (*b*) Comparison between IAPS results and the ‘start of plank’ segment from the plank task. (*c*) Comparison between IAPS results and the ‘end of plank’ segment from the plank task. Note that the IAPS results are identical for comparison as the IAPS only had a single recording segment. Error bars represent the standard error of the mean (s.e.m.).

For the first recording segment (elevator), the repeated-measures 2 × 2 × 2 ANOVA with the within-participant factors validity (IAPS, plank), valence (neutral, negative) and electrode pair (F3/4, F7/8) and AIs as dependent variables, there was a main effect of electrode pair (*F*_1,74_ = 4.93, *p* = 0.03, $\eta_{p}^{2}=0.06$). Further, a significant interaction between validity, valence and electrode pair was found (*F*_1,74_ = 5.84, *p* = 0.02, $\eta_{p}^{2}=0.07$). No *post hoc* test reached significance, however, after applying a Bonferroni correction (all *p*s > 0.12) (figure 5*a*).

Analysis of the second recording segment (start of plank) revealed a main effect of electrode pair (*F*_1,74_ = 6.86, *p* = 0.01, $\eta_{p}^{2}=0.09$). A threefold interaction between validity, valence and electrode pair was found (*F*_1,74_ = 4.11, *p* = 0.046, $\eta_{p}^{2}=0.05$). There was a trend towards significance in the more ecologically valid plank task on the electrode pair F3/4 (*p* = 0.06). Here, the AI decreased from the neutral (*M* = 0.09, s.d. = 0.66) to the negative condition (*M* = −0.05, s.d. = 0.27) (figure 5*b*).

The ANOVA for the ‘end of plank’ recording segment showed a main effect of electrode pair (*F*_1,74_ = 5.49, *p* = 0.02, $\eta_{p}^{2}=0.07$). Moreover, the interaction between validity, valence and electrode pair reached significance (*F*_1,74_ = 4.2, *p* = 0.04, $\eta_{p}^{2}=0.05$), but *post hoc* tests did not survive Bonferroni correction. However, there was a trend towards significance that showed the same direction as for the second time point. Within the plank task, we found that AIs decreased from the neutral (*M* = 0.06, s.d. = 0.63) to the negative condition (*M* = −0.08, s.d. = 0.31) on the F3/4 electrode pair (*p* = 0.051) suggesting greater left-hemispheric alpha power and thus stronger right-hemispheric activation in the negative condition (figure 5*c*).

**Figure 6.**
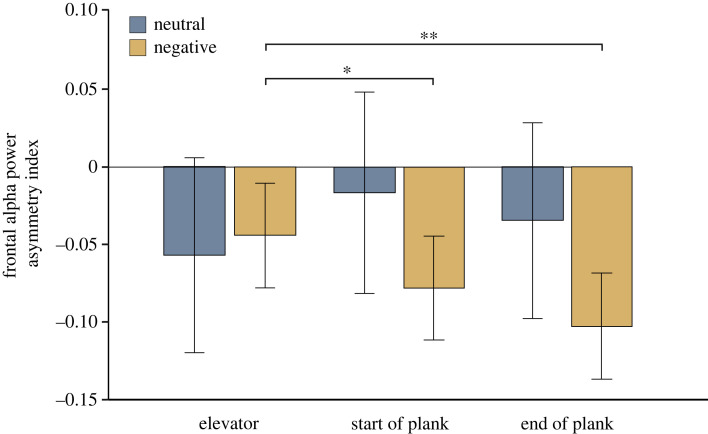
Temporal development of AIs during the plank task in the neutral and negative conditions. In the neutral condition, no changes in AIs were observed over time. In the negative condition, significant decreases in AIs were observed in the ‘start of plank’ and ‘end of plank’ segment with respect to the elevator segment. Error bars represent s.e.m. *p < 0.05 and **p < 0.01.

Specifically for the plank task, the 2 × 2 × 3 repeated-measures ANOVA with the within-participant factors valence (neutral, negative), electrode pair (F3/4, F7/8) and recording segment (‘elevator’, ‘start of plank’, ‘end of plank’) revealed a significant interaction between valence and recording segment (*F*_1.74,128.76_ = 7.84, *p* = 0.001, $\eta_{p}^{2}=0.10$). Bonferroni-corrected *post hoc* tests showed two significant differences within the negative condition pooled across the F3/4 and F7/8 electrode pair. The AI decreased significantly (*p* = 0.01) from ‘elevator’ (*M* = −0.05, s.d. = 0.3) to ‘start of plank’ (*M* = −0.08, s.d. = 0.3) (*p* = 0.012) and from ‘elevator’ to ‘end of plank’ (*M* = −0.11, s.d. = 0.3) (*p* = 0.002). This indicates increasing involvement of the right hemisphere in the course of the negative plank condition (figure 6). The difference between ‘start of plank’ and ‘end of plank’ did not reach significance (*p* = 0.23). No significant differences between time points were found in the neutral condition (all *p*s > 0.11).

#### 3.1.2. Hypotheses 3 and 4

**Figure 7.**
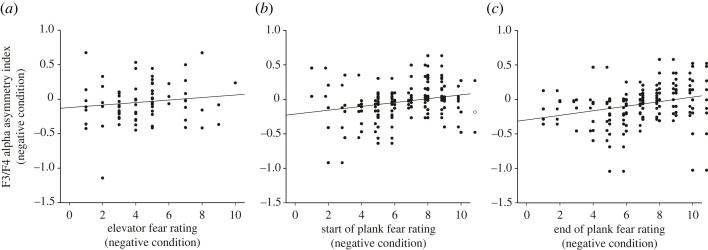
Pearson correlations between AIs at the F3/F4 electrode sites and subjective fear ratings across the recording segments. No significant association was detected between AIs and fear ratings during the (*a*) ‘elevator’ segment. Significant positive correlations were observed for the (*b*) ‘start of plank’ and (*c*) ‘end of plank’ segments.

AIs of the second (‘start of plank’: *r* = 0.23, *p* = 0.045) and third (‘end of plank’: *r* = 0.27, *p* = 0.02) recording segment of the negative plank condition were positively correlated with subjective fear ratings at these recording segments but only for the electrode pair F3/4 (figure 7). This implies that participants with greater subjective fear showed increasingly leftward frontal activity at these electrode sites. Correlations of AIs at the electrode pair F7/8 were not significant (all *p*s > 0.31). Concerning neutral conditions, none of the correlations reached significance (all *p*s > 0.15).

**Figure 8.**
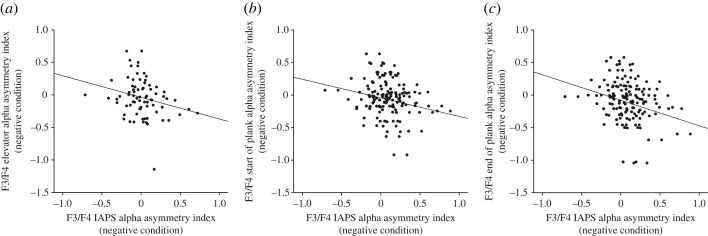
Pearson correlations between AIs at the F3/F4 electrode sites during the IAPS task and across the recording segments. Significant negative correlation coefficients could be detected during the (*a*) ‘elevator’, the (*b*) ‘start of plank’ and the (*c*) ‘end of plank’ segments.

AIs in the negative IAPS condition were negatively correlated with the AIs in the negative plank condition on the electrode pair F3/4 (‘elevator’: *r* = −0.25, *p* = 0.03; ‘start of plank’: *r* = −0.23, *p* = 0.046; ‘end of plank’: *r* = −0.28, *p* = 0.01) (figure 8). Thus, stronger right-hemispheric activation during VR was actually associated with stronger left-hemispheric activation during the IAPS task. There were no significant correlations between the two neutral conditions of the plank and the IAPS task (all *p*s > 0.12).

### 3.2. Exploratory analyses

#### 3.2.1. Self-report data

**Figure 9.**
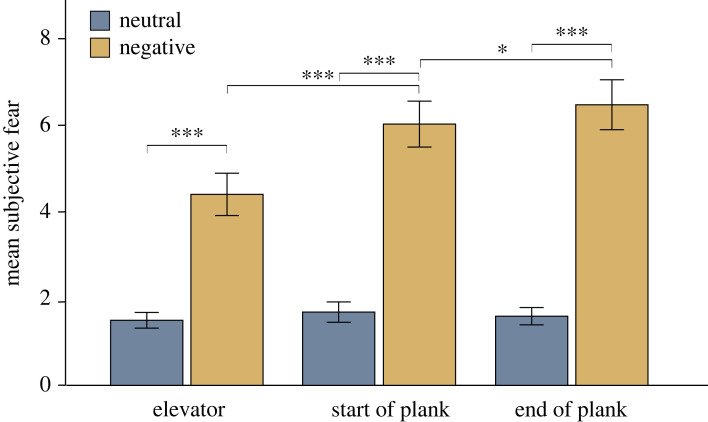
Subjective fear ratings for the neutral and negative condition across the recording segments. Subjective fear ratings did not change across the neutral condition and were at low levels overall. Fear ratings were substantially higher in the negative condition and successively increased over time. Error bars represent s.e.m. *p < 0.05, **p < 0.01 and ***p < 0.001.

Subjective fear ratings in the plank task were analysed with a 2 × 3 repeated-measures ANOVA with the within-participant factors valence (neutral, negative) and recording segment (‘elevator’, ‘start of plank’, ‘end of plank’) (for mean ratings, see electronic supplementary material, table S2). A significant main effect of both valence (*F*_1,73_ = 286.97, *p* < 0.001, $\eta_{p}^{2}=0.8$) and recording segment (*F*_1.58,115.41_ = 72.93, *p* < 0.001, $\eta_{p}^{2}=0.5$) could be detected. Additionally, the factor valence interacted with the factor recording segment (*F*_2,146_ = 53.78, *p* < 0.001, $\eta_{p}^{2}=0.42$). Exclusively in the negative plank condition, the difference in fear was significant between all three recording segments. Thus, self-reported fear successively increased from ‘elevator’ (*M* = 4.41, s.d. = 2.1) to ‘start of plank’ (*M* = 6.03, s.d. = 2.28) (*p* < 0.001), from ‘start of plank’ to ‘end of plank’ (*M* = 6.47, s.d. = 2.47) (*p* = 0.02), and from ‘elevator’ to ‘end of plank’ (*p* < 0.001) (figure 9). At every recording segment, subjective fear ratings were greater in the negative than in the neutral plank condition (all *p*s < 0.001).

Correlations between fear ratings and the questionnaires were calculated. There was no significant correlation between the STAI-T score and self-reported fear regardless of condition (all *p*s > 0.33). The STAI-S was positively correlated with the ‘end of plank’ rating of both the neutral (*r* = 0.29, uncorrected *p* = 0.01, corrected *p* = 0.279) and the negative plank task (*r* = 0.33, uncorrected *p* < 0.01, corrected *p* = 0.096) and with the ‘start of plank’ rating in the negative condition only (*r* = 0.24, uncorrected *p* = 0.04, corrected *p* = 0.936). Thus, participants with higher state values of anxiety before the experiment later reported higher fear in the ratings especially in the negative condition. Specifically for fear of heights, the acrophobia and the avoidance subscale of the ACRO/AVOI questionnaire were positively correlated with all three ratings (table 1) of the negative plank condition. The acrophobia subscale was further significantly correlated with the ‘elevator’ and the ‘end of plank’ rating of the neutral condition. However, these correlations did not survive Bonferroni correction.

**Table 1.** Correlations of fear ratings in the plank conditions with pre- and post-questionnaires. *Note*. Bonferroni-corrected significant values (*p* = 0.0021) are printed in italics.

	plank (neutral)			plank (negative)		
	elevator	start of plank	end of plank	elevator	start of plank	end of plank
acrophobia (ACRO/AVOI)	0.25*	0.2	0.27*	0.46***	0.49***	0.51***
avoidance (ACRO/AVOI)	0.14	0.1	0.15	0.31**	0.38***	0.4***
presence (IPQ_G)	0.05	−0.06	−0.03	0.4***	0.39***	0.34**
simulator sickness (SSQ)	0.12	0.11	0.16	0.28*	0.33**	0.32**

*p ≤ 0.05, **p ≤ 0.01, ***p ≤ 0.001.

Fear ratings of the neutral plank task did not correlate with any of the presence ratings (all *p*s > 0.7). Correlations of the fear ratings in the negative plank task with corresponding presence ratings reached significance at every recording segment (‘elevator’: *r* = 0.47, *p* < 0.001; ‘start of plank’: *r* = 0.57, *p* < 0.001; ‘end of plank’: *r* = 0.55, *p* < 0.001). Thus, participants with higher self-reported presence also indicated higher fear. Overall presence ratings can be found in electronic supplementary material, table S2.

Correlations of fear ratings with post-experimental questionnaires (see electronic supplementary material, table S3 for descriptive statistics) yielded significant positive correlations between all the subscales of the presence questionnaire (IPQ) and subjective presence ratings during the experiment, regardless of condition (see tables 1 and 2). Participants who stated a more pronounced feeling of presence during the experiment showed higher values in the IPQ which was filled out after the experiment. Significant correlations were found between two of the fear ratings in the negative plank condition (‘start of plank’ and ‘end of plank’) and the SSQ score (table 1). Higher subjective fear at these recording segments was accompanied by more pronounced physical symptoms (e.g. nausea, dizziness). After Bonferroni correction, these correlations were, however, not significant any more.

**Table 2.** Correlations between presence ratings in the plank task and the subscales of the IPQ. *Note*. Bonferroni-corrected significant values (*p* = 0.0021) are printed in italics. G, general item; SP, spatial presence; INV, involvement; REAL, experienced realism. IPQ_G is an additional general item and does not count as a subscale of the IPQ. IPQ_SP measures the feeling of being in the simulation. IPQ_INV enquires how focused and involved participants were in the VR. IPQ_REAL represents how real the VR experience felt.

	plank (control)			plank (experimental)		
	elevator	start of plank	end of plank	elevator	start of plank	end of plank
presence (IPQ_G)	0.42***	0.48***	0.5***	0.56***	0.58***	0.39***
presence (IPQ_SP)	0.4***	0.48***	0.52***	0.55***	0.51***	0.26*
presence (IPQ_INV)	0.26*	0.32**	0.31**	0.38***	0.4***	0.24*
presence (IPQ_REAL)	0.45***	0.5***	0.5***	0.57***	0.44***	0.36***

*p ≤ 0.05, **p ≤ 0.01, ***p ≤ 0.001.

The repeated-measures 3 × 2 ANOVA of the PANAS questionnaires with the within-participant factors time (baseline, post-VR, post-IAPS) and affect (positive affect, negative affect) showed significant main effects of time (*F*_1.78,131.86 =_ 53.63, *p* < 0.001, $\eta_{p}^{2}=0.42$) and affect (*F*_1,74_ = 391.77, *p* < 0.001, $\eta_{p}^{2}=0.84$). A significant interaction of time and affect was found (*F*_1.64,121.26_ = 85.84, *p* < 0.001, $\eta_{p}^{2}=0.54$). *Post hoc* Bonferroni correction for the main effect of time showed a significant increase of positive and negative affect from baseline (*M* = 1.86, s.d. = 0.27) to post-VR (*M* = 2.17, s.d. = 0.28) (*p* < 0.001) and a decrease from post-VR to post-IAPS (*M* = 1.82, s.d. = 0.36) (*p* < 0.001). *Post hoc* Bonferroni tests of affect revealed that participants generally reported higher positive affect (*M* = 2.59, s.d. = 0.46) than negative affect (*M* = 1.31, s.d. = 0.25) (*p* < 0.001). After *post hoc* Bonferroni correction of the time × affect interaction, results showed that positive affect increased from baseline (*M* = 2.6, s.d. = 0.5) to post-VR (*M* = 3.02, s.d. = 0.48) (*p* < 0.001) and then decreased at the post-IAPS time point (*M* = 2.15, s.d. = 0.64) (*p* < 0.001). The decrease of positive affect between baseline and post-IAPS also reached significance (*p* < 0.001). For negative affect, there was a successive increase from baseline (*M* = 1.13, s.d. = 0.2) to post-VR (*M* = 1.32, s.d. = 0.29) (*p* < 0.001) to post-IAPS rating (*M* = 1.49, s.d. = 0.52) (*p* = 0.01). The difference between baseline and post-IAPS was also significant (*p* < 0.001).

#### 3.2.2. Movement signals

Additional multiple linear regressions were conducted to investigate the predictive value of movements (*X*-, *Y*- and *Z*-axes) on the AIs. According to the Durbin–Watson statistic, residuals were independent. Approximate normality was checked via P-P-plots and histograms of the standardized residuals. Homoscedasticity and linearity were indicated based on the scatterplot of the residuals. According to the VIF, multi-collinearity was not of concern (for the neutral condition: all VIF < 2; for the negative condition: all VIF < 2.77). In the neutral condition of the plank task, movements predicted AIs on the electrode pair F3/4 at two different recording segments (‘start of plank’: *F*_3,70_ = 3.63, *p* = 0.02, *R*^2^ = 0.13, ‘end of plank’: *F*_3,71_ = 2.83, *p* = 0.045, *R*^2^ = 0.11) (electronic supplementary material, table S4). After further analysis, only the *X*-axis significantly predicted AIs in the neutral plank task at these recording segments. No further significant influences of movement sensors could be detected (all *p*s > 0.1; see electronic supplementary material, table S5 for results in the negative plank condition). A significant influence of movements on the electrode pair F3/4 was found for the neutral IAPS task (*F*_3,71_ = 4.69, *p* = 0.001, *R*^2^ = 0.165) (electronic supplementary material, table S6). The model can explain 16.5% of the variance in the AIs of the neutral IAPS task. However, only the *X*-axis constituted a significant predictor (F3/4: *p* = 0.001). All regression models can be found in electronic supplementary material, tables S4–S6.

## 4. Discussion

We aimed to investigate the neural correlates of fear processing both in an immersive and a two-dimensional pictorial setting. In the immersive design, a virtual height paradigm was combined with a mobile EEG system to assess hemispheric asymmetries. These were compared with brain asymmetry patterns in response to two-dimensional emotional IAPS images to draw conclusions about the ecological validity of the established emotion induction method.

### 4.1. Behavioural results during virtual reality

We found clear indications that the fear induction of the VR paradigm worked exceptionally well using subjective ratings. Although research should not rely solely on subjective measures to evaluate emotion induction, fear ratings have generally been proven to be valuable ([67]; for a review, see [68]). Indeed, our results revealed substantially higher fear ratings in the negative VR condition compared with the neutral condition throughout all recording segments. Additionally, ratings showed an incremental increase in subjective fear over time. Thus, at least on a subjective level, participants experienced a considerable growth in fear when standing on the heightened plank. Notably, these patterns were not found in the neutral plank walk on the ground. Thus, the plank paradigm is a valid tool to induce fear not only between different conditions (negative versus neutral) but also within conditions, as standing within the safety of the elevator is perceived as less fearful compared with standing at the end of the plank.

### 4.2. Hypothesis 1: alpha asymmetries in the virtual reality task

The first hypothesis targeted the efficacy of the VR paradigm in inducing fear reflected by altered hemispheric asymmetries in response to the virtual height simulation. Drawing on research on lateralization theories, higher left-hemispheric alpha power (reflecting greater right-hemispheric activity) in the negative compared with the neutral VR condition was expected [69,70]. In the present study, the AI on electrode pair F3/4 indicated greater activation of the right hemisphere in the negative plank condition for the ‘start of plank’ and the ‘end of plank’ recording segment compared with the neutral condition but only on a trend level. This is in line with a VR study by Rodríguez Ortega *et al.* [71]. They reported that right-hemispheric alpha band activity decreased in response to VR-induced sad mood, supporting rightward dominance in negative emotion processing. The trends in the present experiment imply that there was a difference in brain activation between the neutral and the negative plank condition but only when standing on the plank when fear ratings were highest. Since these results were not statistically significant, we need to treat this interpretation with caution, and these results should be replicated in the future to gain more confidence in them. The notion of an increase in right-hemispheric activation in response to negative affect, however, is supported by the change in AIs in the negative plank condition as they successively shifted leftward indicating increasing involvement of the right hemisphere. This development was only significant between the first and the second and between the first and the third recording segments. Apparently, looking down the abyss from inside the elevator was not as powerful in evoking fear as standing on the plank. The time-dependent tendency towards right-hemispheric processing is well in line with the self-reported fear ratings as they were at the lowest in the elevator and successively increased on the plank.

### 4.3. Hypothesis 2: alpha asymmetries in the international affective picture system task

The second hypothesis predicted more negative AIs in the negative IAPS condition compared with the neutral IAPS condition. Surprisingly, no significant differences in brain asymmetries were found between the neutral and the negative condition. Despite the widespread use of the IAPS, findings about emotion-related hemispheric asymmetries elicited by the images remain controversial. For positive emotions, Gable & Harmon-Jones [72] found leftward activation to appetitive two-dimensional stimuli. Alterations of brain asymmetry in multiple brain regions during IAPS exposure were also reported by Orgo *et al.* [73]. At the same time, various studies have failed to detect asymmetric brain activation patterns during emotional picture presentation [74–76]. A possible explanation for this inconsistency in the literature and the null results in our data could be attributed to a lack of emotional impact of the IAPS images hindering naturalistic emotional brain responses. Another possible explanation could relate to the experience of the highly emotional VR task prior to the IAPS task. Having experienced the simulation beforehand could have had an effect on the IAPS task, rendering the latter subjectively less threatening.

### 4.4. Hypothesis 3: relationship between fear ratings and alpha asymmetries in the plank task

The third hypothesis assumed a negative correlation between fear ratings and AIs in the negative plank condition. In the neutral condition, no significant correlations were revealed. For the ‘start of plank’ and ‘end of plank’ recording segment of the negative plank condition, however, there were positive correlations between fear ratings and AIs indicating that greater fear was associated with greater left-hemispheric activation. The results were thus in the opposite direction from what we hypothesized. We can only speculate about the nature of this unexpected finding. One potential explanation for the enhanced left-hemispheric activation in participants with higher self-reported fear could be the application of emotion regulation strategies. Goodman *et al.* [77] found more pronounced leftward frontal asymmetry in particularly stressful situations. This activation pattern predicted better emotion regulation in participants and was accompanied by higher self-reported stress and anxiety. Similar findings were obtained by Berretz *et al.* [78] who recorded EEG signals during the Trier Social Stress Test. If participants in the present study suppressed their emotions, applied cognitive re-appraisal, or distracted themselves from standing on the plank, the EEG signal could be biased towards regulatory brain activation rather than reflecting a pure correlate of fear [79]. Thus, participants reporting higher self-reported fear could have applied emotion regulation strategies requiring greater left-hemispheric frontal involvement.

### 4.5. Hypothesis 4: comparison of alpha asymmetries between tasks

The fourth hypothesis aimed at testing the ecological validity of the tasks. Ecological validity was postulated to differ significantly between the emotion induction methods used in this study, positive correlations were assumed between AIs of the negative IAPS condition and the negative plank condition. Higher correlations were expected between both neutral conditions. However, the data did not confirm this hypothesis as no correlations were detected between neutral conditions, and we found low negative correlations for all three recording segments in the negative conditions. Since alpha oscillations show acceptable short-term reliability [80], it is unlikely that this result is due to spurious changes in oscillatory activity. It is possible that using a more naturalistic paradigm activates different brain networks, as patterns between the two tasks were only weakly related, in both negative and neutral settings.

### 4.6. Movement signals

In this study, the impact of movements on the EEG signal was tested with multiple linear regressions. Results revealed significant effects implying a predictive value of movements on the EEG signal. However, these effects occurred only in the neutral conditions of both tasks and solely on electrode pair F3/4. A systematic impact of movement should have been evident consistently across conditions and recording segments. Similarly, several studies have demonstrated that movements do not significantly influence the EEG signal in mobile EEG- or VR-based tasks [44,46,49]. Especially in asymmetry research, the use of a relative measure between hemispheres probably cancels out any residual movement artefacts. Thus, the selective findings between movements and the EEG signal in our study seem to be coincidental rather than systematic.

### 4.7. Limitations and future directions

While in our study we aimed to provide a highly naturalistic paradigm, one might reasonably argue that walking atop a plank on a skyscraper is not a realistic scenario. Even though fear and presence ratings were generally high, they could be further improved by implementing a variety of sensory cues. Previous studies [81,82] found a positive relationship between multi-modal sensory stimulation and sense of presence, in that a higher range in stimulated senses during VR resulted in a higher sense of presence. Especially auditory stimuli corresponding to the simulated situation seem to create ‘realness’ [82]. In this experiment, auditory cues such as street noise or elevator noises could be implemented via headphones to create a more realistic surrounding. Additionally, a fan could give the impression of wind when leaving the elevator adding a somatosensory dimension. Thus, future studies could try to introduce a broader variation of sensory stimulation to enhance immersion into the VR.

As opposed to established fear induction methods such as images or films, the use of VR systems in experiments entails different challenges on a technological level (for advantages and disadvantages of different VR systems, see [83]). In combining EEG with VR systems, concerns have been raised that the latter could interfere with signal transmission especially in experimental tasks involving extensive movement. However, it was shown that at least on an electrophysiological level, this seems to be a negligible risk [84]. But while VR opens further possibilities in experimental emotion induction, standardized requirements for the technical framework (e.g. resolution parameters) are not yet set. Therefore, it is necessary to assess variables such as simulator sickness or system usability to address the higher not only emotional but also physical impact of VR. Another issue regarding technical realization is body ownership within VR. In the present study, participants only saw their virtual hands to operate the elevator but not the rest of the body. This could have reduced presence [83] and, therefore, the fear-inducing effect of walking on the plank.

In terms of measurements, there are possible improvements in the behavioural as well as in the physiological dimension. Since we speculated that emotional regulation might have played a role in the observed result pattern, a questionnaire assessing emotion regulation strategies (e.g. the German version of the Emotion Regulation Questionnaire [85]) would have been helpful to provide further support for this notion. On a physiological level, a complementary parameter recording peripheral responses to emotional material could also have been implemented. As discussed above, heart rate (or heart rate variability) and skin conductance constitute promising candidates for future research [86,87]. Finally, eye tracking could be considered as an additional measure to control for gaze direction. During the EEG recording on the plank, participants were instructed to freely explore their virtual surrounding to enable as much naturalistic behaviour as possible. Therefore, control over the extent to which participants looked down the abyss was limited, and a certain amount of height avoidance was possible. This could be addressed by monitoring the participants' gaze. Fortunately, this feature can easily be included in the VR system without requiring further equipment. This paradigm would also be highly suitable in the context of clinical laterality research to identify if, for example, phobic patients show altered lateralization patterns compared with healthy controls [88]. Furthermore, the reliable fear induction could also indicate that this paradigm is a suitable stress induction paradigm to study laterality patterns in more naturalistic settings.

All in all, the paradigm used in this study holds much potential for future laterality research. For example, as Mundorf & Ocklenburg [88] point out, there are many open questions on the role of lateralization in psychiatric and neurodevelopmental disorders. Thus, the field of clinical neuroscience could certainly benefit from a more naturalistic approach to the investigation of hemispheric asymmetries. Beyond laterality research, the present paradigm could provide a promising alternative to classical stress paradigms as the participants reported high levels of fear that are typically related to stress responses [89]. Future studies could thus measure endocrinological responses in response to the negative and neutral condition to validate their stressfulness. To study the effects of emotion regulation further, one could also adopt a re-appraisal paradigm in which participants receive guided instructions how to regulate the negative affect on the plank by, for example, reminding themselves of the virtual simulation.

## 5. Conclusion

VR technology is already widespread in clinical research, especially in the treatment of anxiety disorders, and has proven to be effective on a behavioural level [25,90–93]. This study provides evidence that also the corresponding neurophysiological correlates of emotional processing are more reliably assessed in naturalistic settings that allow for feeling and corresponding action [94]. The combination of mobile EEG and VR could thus shed light on prevailing theories of emotional lateralization such as the right hemisphere and valence hypothesis in the future, as this topic is still heavily debated.

## Data Availability

All data and analyses are accessible publicly at OSF under the following link: https://osf.io/vzjrk/files/osfstorage#. The data are provided in the electronic supplementary material [95].
